# Multivariate Optimization for Extraction of Pyrethroids in Milk and Validation for GC-ECD and CG-MS/MS Analysis

**DOI:** 10.3390/ijerph111111421

**Published:** 2014-11-05

**Authors:** Leonardo Zanchetti Meneghini, Gabriel Rübensam, Vinicius Claudino Bica, Amanda Ceccon, Fabiano Barreto, Marco Flores Ferrão, Ana Maria Bergold

**Affiliations:** 1 Laboratório Nacional Agropecuário, Ministério da Agricultura, Pecuária e Abastecimento, Estrada da Ponta Grossa, 3036 Porto Alegre, RS 91785-340, Brazil; E-Mails: grubensam@yahoo.com (G.R.); vinibicca@yahoo.com.br (V.C.B.); amanda_ceccon@hotmail.com (A.C.); fabiano.barreto@agricultura.gov.br (F.B.); 2 Faculdade de Farmácia, Universidade Federal do Rio Grande do Sul, Av. Ipiranga, 2752 Porto Alegre, RS 90610-000, Brazil; E-Mail: ana.bergold@ufrgs.br; 3 Instituto de Química, Universidade Federal do Rio Grande do Sul, Av. Bento Gonçalves, 9500 Bairro Agronomia, Porto Alegre, RS 91501-970, Brazil; E-Mail: mfferrao@gmail.com

**Keywords:** Doehlert design, GC-MS/MS, GC-ECD, pyrethroids, bovine milk

## Abstract

A simple and inexpensive method based on solvent extraction followed by low temperature clean-up was applied for determination of seven pyrethroids residues in bovine raw milk using gas chromatography coupled to tandem mass spectrometry (GC-MS/MS) and gas chromatography with electron-capture detector (GC-ECD). Sample extraction procedure was established through the evaluation of seven different extraction protocols, evaluated in terms of analyte recovery and cleanup efficiency. Sample preparation optimization was based on Doehlert design using fifteen runs with three different variables. Response surface methodologies and polynomial analysis were used to define the best extraction conditions. Method validation was carried out based on SANCO guide parameters and assessed by multivariate analysis. Method performance was considered satisfactory since mean recoveries were between 87% and 101% for three distinct concentrations. Accuracy and precision were lower than ±20%, and led to no significant differences (*p* < 0.05) between results obtained by GC-ECD and GC-MS/MS techniques. The method has been applied to routine analysis for determination of pyrethroid residues in bovine raw milk in the Brazilian National Residue Control Plan since 2013, in which a total of 50 samples were analyzed.

## 1. Introduction

**Figure 1:**
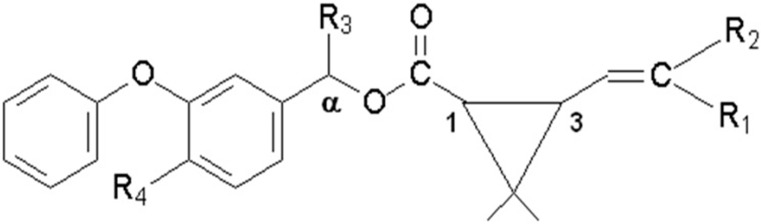
General structure of the pyrethroids included in the study.

Currently, parasiticide drugs are considered one of the pillars that sustain the extensive livestock, particularly in tropical regions where the environmental conditions, such as high temperature and humidity, have an important role on both cattle production and the spread of parasites in farm animals. In Brazil, which is one of the world's largest producers of food of animal origin, parasitic diseases are by far the most important factor responsible for livestock production losses [1]. Among all parasites that affect livestock in Brazil, ectoparasites such as ticks have been considered as one of the main causes of substantial reductions in the production of food such as milk [2]. Nowadays, the control of parasitic diseases of veterinary importance still relies on the use of chemicals, being pyrethroid-insecticides (PYR; Figure 1 and Table 1), especially cypermethrin, widely used for this purpose [2,3,4]. Although most parasites have a well-defined life cycle, which leads to the application of antiparasitic drugs only in specific periods along the year, tick infestations may occur at any time and control relies on the continuous usage of PYR in dairy cattle, which may lead to the undesirable occurrence of its residues in milk or meat [5,6,7,8].

**Table 1:** Substituted radicals represented in the Figure 1 for each pyrethroid.

Pyrethroid	MW ^a^	R1 ^b^	R2 ^b^	R3 ^b^	R4 ^b^
Gamma-cyhalothrin	449.9	Cl	CF_3_	CN	H
Lambda-cyhalothrin	449.9	Cl	CF_3_	CN	H
cyfluthrin	391.3	Cl	Cl	H	H
cypermethrin	434.4	Cl	Cl	CN	F
permethrin	391.3	Cl	Cl	CN	H
deltamethrin	434.4	Br	Br	CN	H
fenvalerate	416.3	H	Cl	CN	H

Notes: ^a^ molecular weight; ^b^ susbtituted radical represented in the Figure 1.

There are several ways these compounds reach the milk, including improper use of the drug, contamination of animal feedstuffs, environmental contamination (from the use of PYR as insecticide) and animal-to-animal drug transfer compromising milk quality. Moreover, considering the diverse nature of PYR contamination, this group has been studied in both environment and food contamination cycles [4,5,6,7].

According to recent reports, PYR has mainly been analyzed in different matrices either by liquid chromatography with UV detection (LC-UV) or coupled to mass spectrometry with single (LC-MS) or triple quadrupole (LC-MS/MS), and by gas chromatography with electron capture detector (GC-ECD) or coupled to mass spectrometry with single (GC-MS) or triple quadrupole (GC-MS/MS). Although a greater emphasis has been given to mass spectrometry detectors due to the confirmatory nature of this technique, GC-ECD is an important routine tool and the most commonly used detection technique for PYR analyses at low detection limits [4,9,10,11,12,13,14]. However, methods based on GC-ECD commonly require more laborious and complex sample preparation in order to remove matrix compounds that can improve the imprecision of the analytical method. Furthermore, it can lead to an increase in the use of sorbents and organic solvents.

In Brazil, PYR residues in milk are monitored by a network of analytical laboratories (LANAGRO) through the National Residue Control Plan (NRCP) established by the Brazilian Ministry of Agriculture (MAPA). Generally, PYR residues were determined using methods based on extraction with C18 sorbents and analysis by gas chromatography. Private laboratories linked to MAPA laboratories network may use other analytical methods since they are sensible, confirmatory, and has been fully validated. To meet these requirements for the analysis of PYR in complex matrices, it is essential that the sample preparation technique be effective and provide extracts as free as possible of interfering compounds. For this purpose, different sample extraction procedures have been employed based on techniques such as liquid-liquid extraction (LLE) [15], solid-phase extraction (SPE), solid-phase micro extraction (SPME) [16], matrix solid-phase dispersion (MSPD) [17], supercritical fluid extraction (SFE) [18] and others, typically in off-line mode and, more recently, in on-line procedures and also by using automatic devices [19]. The advantages and drawbacks of each technique must be considered. For instance, the combination of high temperature and pressure (SFE) may cause degradation and/or isomer conversion of synthetic pyrethroids; presence of carry-over effect or high cost (SPME) [19]. Recently, our laboratory (LANAGRO-RS) has proposed an alternative cleanup procedure based on low temperature cleanup (LT) instead of sorbent-based procedures for residue analysis in milk [20]. Briefly, raw extract was put on a freezer at −20 °C [14]. Under this condition, the interfering compounds are frozen, whereas moderately polar to apolar analytes remains in the liquid phase and are subsequently separated [14].

Variability in the matrix composition plays important role in GC-ECD analysis because co-extractive compounds can generate variations in instrumental response (e.g., liner interaction, baseline noise) and, in this way, the extraction method requires a carefully planned optimization. Notwithstanding, fat content and other endogenous compounds are subject to seasonal and regional variation. Besides, some physico-chemical PYR characteristics, including lipophilicity and surface adsorption, can generate inefficient extraction rates [19,21,22].

Method development became more effective using multivariate optimization because more information is obtained about the interaction among the variables, sometimes undetected when univariate approach is used [19,23,24,25]. For analytical purposes, the Doehlert one has shown to be the most adequate kind of design and can be applied to response surfaces with a good estimation of the parameters of the quadratic mathematical model, allowing the study of independent variables, at different levels, and has been successfully used for optimization of extraction methods in food analysis [25,26].

In this way, the present work describes the use of a Doehlert design to optimize the development of a low cost method for determination of the pyrethroids cyfluthrin (CYF), gamma-cyhalothrin (g-CYH), lambda-cyhalothrin (l-CYH), cypermethrin (CYP), deltamethrin (DEL), fenvalerate (FEV) and permethrin (PER) in raw milk using GC-ECD and GC-MS/MS, as well as the practical issues for the implementation of the proposed method in the Brazilian NRCP.

## 2. Experimental Section

### 2.1. Chemicals and Apparatus

Individual PYR standards with purity between 96.7%–99.8% (CYF, CYP, l-CYH, g-CYH, DEL, FEV and PER) were obtained from Riedel-de-Häen (Seelze, Germany). CYF, CYP, FEV and PER were a mixture of isomers. A stock solution of each pesticide was prepared individually in acetonitrile (ACN) to obtain the primary calibration solution (1000 mg·L^−1^; stored at −20 °C) from which the intermediate standard solutions were prepared by dilution in ACN at 100 mg·L^−1^ and stored in a refrigerator at 5 °C. Methanol (MeOH), hexane (HEX), acetone (ACO), ethyl acetate (EAC), anhydrous sodium sulfate (Na_2_SO_4_), were purchased from Mallinckrodt Baker (Phillipsburg, NJ, USA). The sorbent material for the matrix solid phase dispersion (MSPD) was Lichroprep^®^ RP-C18 (25–40 μm, non-endcapped, 16% carbon load; Merck^®^, Darmstadt, Germany) that was exhaustively prewashed with solvent including MeOH, ACO, ACN and HEX before use. Blank milk samples were obtained from certified producer. Raw milk samples were collected in dairy plants by Federal Inspection Service (SIF).

### 2.2. Chromatographic Analysis

GC-ECD analysis was achieved using a GC Trace Ultra (Thermo^®^) gas chromatograph equipped with a split/splitless injection system (used in splitless mode), autosampler AI 3000^®^ and a ^63^Ni ECD source. For separation of PYR, several capillary columns were tested: 14% cyanopropyl-phenyl-methylpolysiloxane (OV-1701, Ohio Valley, 30 m × 0.53 mm × 0.5 μm film thickness), 100% dimethylpolysiloxane (ZB1, Phenomenex, 30 m × 0.25 mm × 0.25 μm film thickness), 5% phenyl-methyl-polysiloxane (OV-5, Ohio Valley, 15.0 m × 0.25 mm × 0.1 μm film thickness) and 5% phenyl-95% dimethyl polisiloxane (ZB5, Phenomenex, 30 m × 0.25 mm × 0.10 μm film thickness). The temperature program for these columns were the same: 100 °C (1 min) to 250 °C at 20 °C·min^−1^, to 260 °C (3 min) at 5 °C·min^−1^ and to 330 °C (5 min) at 20 °C·min^−1^. The injector and detector temperature were 240 °C and 340 °C, respectively. Helium was used as carrier gas with the flow of 1.2 mL·min^−1^ and N_2_ as make-up gas (30 mL·min^−1^). The injection volume was 3.0 μL in splitless mode for all standards and samples. This technique was used for both extraction method evaluation and, after method validation, for real sample analysis.

Analysis by GC-MS/MS was performed in an Agilent 7000 gas chromatograph (Agilent, Santa Clara, CA, USA) coupled to a mass spectrometry detector in tandem mode. Samples were introduce into GC using an auto sampler 7890 A with pulsed splitless mode with temperature program 70 °C at 0.1 min, 400 °C·min^−1^ until 240 °C and pulse of the 35 psi for 3 min; 3 μL. Mass spectrometry analysis was carried out through multiple reaction mode (MRM) monitoring 2 transitions (qualitative and quantitative). Chromatographic column was a DB-5ms (30 m × 0.25 mm × 0.25 μm film thickness). Helium was used as carrier gas with the flow of 1.2 mL·min^−1^ and N_2_ was used as collision gas. This technique was used for real sample analysis after method validation.

### 2.3. Extraction Methodologies

**Figure 2:**
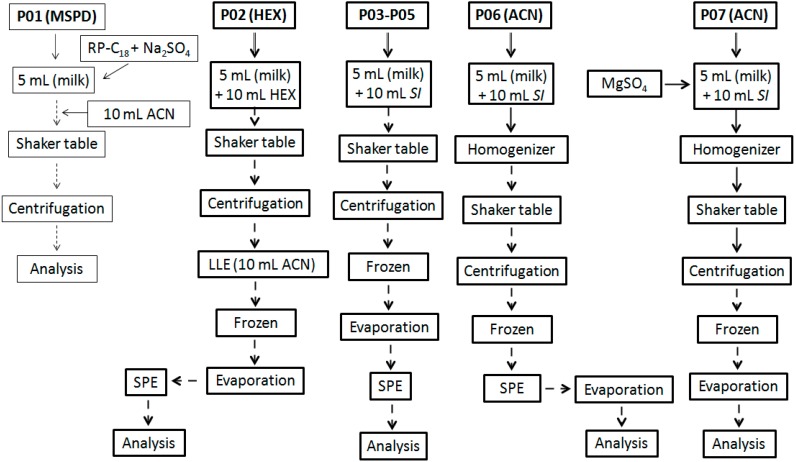
Summary of the procedures evaluated for sample preparation.

Different procedures, named as *protocol* (*P*; numbered from 1 to 7), were used and evaluated in terms of extraction and cleanup efficiency. The workflow summary is showed in the Figure 2 (P01 to P07). They were carried out on six replicates per protocol in batches composed by blank samples spiked with all PYR analytes at maximum residue level (MRL) defined in NRCP 2013 [23,24]. Extraction efficiency was verified in terms of recovery (*RE*; percentage) by comparison between tissue standard (*TS*; samples spiked after extraction procedure, in a matrix-matched approach) and samples spiked with PYR pool before extraction (standard addition method). Additionally, clean-up efficiency was evaluated in terms of remaining co-extractive material (CoE) determined by gravimetry, weighting the sample tube before the introduction of the extract and after the full evaporation of the extract solvent and by spectrophotometric measurements (505 nm, Analisa^®^ assay kit for triglycerides quantification), measuring the absorbance intensity of the extract introduced on a spectrophotometer. The protocols used in the present study are briefly described thereupon.

P01 was based on MSPD approach (Lichroprep^®^, RP-C18, Merck) using a sample volume (SV) of 5 mL, which was subsequently added with 2 g of RP-C18 plus 2 g of Na_2_SO_4_, homogenized in vortex for 1 min and stabilized by 1 h under continuous stirring in a shaker. Then, 10 mL of ACN was added to the mixture in order to produce analyte extraction. This mixture was homogenized for 2 min and centrifuged (15 min, 4000 *g*, 0 °C). The supernatant was transferred for a new tube, evaporated to dryness under N_2_ flow, at 40 °C, and reconstituted with 1.0 mL of ACN.

P02 was based on liquid-liquid extraction (LLE) with purification at low temperature (LLE-LTP). For P02, hexane was used as starting solvent (SI), adding 10 mL to a tube containing milk and homogenizing the mixture in a horizontal table (180 rpm, 30 min). This mixture was subsequently centrifuged and the supernatant was transferred to the new tube and stored in the freezer (−20 °C for 12 h). When raw milk was used as sample, an additional step of LLE with ACN (10 mL) was necessary before the frozen step in order to remove less lipophilic interfering compounds. After 12 h, the clean liquid phase was passed through a glass column containing Na_2_SO_4_ (2.0 g), transferred to a new tube and evaporated to dryness (40 °C) under N_2_ flow. The residual content was reconstituted to 1.0 mL with hexane and washed with a mixture of hexane: ethyl acetate (v/v; 98:2) through a silica SPE cartridge. Eluate was evaporated to dryness (40 °C) under N_2_ flow, reconstituted to 300 μL (ACN) and analyzed by GC. The protocols P03-P05 employed the same workflow just changing the SI for acetone, ethyl acetate, and ACN (P03, P04 and P05, respectively).

The P06, after solvent addition, a high-speed homogenizer (Ultra-Turrax^®^; 5 s, 4000 rpm) was used as additional operation but maintaining the same others steps used in P02–P05. Extraction with P07 followed the same workflow that P06, but before high-speed homogenizer step, MgSO_4_ was added, which dismissed the subsequent use of SPE.

### 2.4. Experimental Optimization

The protocol that showed the best results for recovery in initial screening was optimized using a Doehlert design. This kind of multivariate strategy use a number of experiments (N) defined by the equation N = k^2^ + k + 1, where k is the number of factors (variables) involved in the study [19,20]. Least squares were carried out to generate first and second degree polynomials. The adjustment was evaluated by variance analysis (regression, lack-of-fit, residual, pure error) and *t*-test for individual coefficients and respective interactions. Visual inspection of response surface was performed to find a stationary ridge, a simple maximum/minimum, a saddle point (minimax) or a simple maximum (first order polynomial), when applicable. 

For each surface obtained, one variable was fixed in its optimum response (maximum recovery) and applied to next surface returning for the first to compare factors levels and optimum response. When a factor was not considered significant, a central point (level 0 of experimental design) was chosen to practical criteria of robustness [23,24]. Thus, the optimal conditions for extraction were evaluated using a three-level factorial design with a central point. Procedure P07 was chosen to be optimized using agitation-time (*t*; minutes), ACN extraction volume (*V*; mL) and homogenization time (Ultra-turrax^®^; *H*; seconds) as variables to build Doehlert design. The response evaluated was recovery (R%) for each analyte. The design with all variables and respective codes are showed in Table 2. The number of experiments carried out was 15 (three replicates at central point to appropriate number of degree of freedom). A Doehlert design (Doehlert 1.0) developed at Laboratório de Quimiometria Teórica e Aplicada of UNICAMP/Brazil and MATLAB R2012a (Mathworks^®^, Natick, MA, USA) were used for chemometric analysis.

**Table 2:** Doehlert matrix for the variables used in sample preparation optimization.

Experiment ^a^	Coded Values ^b^			Planned Values ^c^		
	*t*	*V*	*H*	Agitation Time (min)	ACN (mL)	Homogenization Time (s)
1	1	0	0	30	10.5	10.0
2	0.5	0.866	0	25	13.5	10.0
3	0.5	0.289	0.817	25	11.5	14.4
4	−1	0	0	10	10.5	10.0
5	−0.5	−0.866	0	15	7.5	10.0
6	−0.5	−0.289	−0.817	15	9.5	5.6
7	0.5	−0.866	0	25	7.5	10.0
8	0.5	−0.289	−0.817	25	9.5	5.6
9	−0,5	0.866	0	15	13.5	10.0
10	0	0.577	−0.817	20	12.5	5.6
11	−0.5	0.289	0.817	15	11.5	14.4
12	0	−0.577	0.817	20	8.5	14.4
13	0	0	0	20	10.5	10.0
14	0	0	0	20	10.5	10.0
15	0	0	0	20	10.5	10.0

Notes: ^a^ Experiments performed in random order with number *13* until *15* used as central points of design; ^b^ Codes *t*, *V*, *H* correspond to *Agitation time*, *ACN*, *Homogenization time*, respectively; ^c^ *ACN* is the solvent employed as *starting solvent* in the procedure.

### 2.5. Validation Procedure

The best conditions found by Doehlert design for P07 were used to perform method validation. Validation level (VL) used for all pyrethoids was 25 μg·mL^−1^ based on the lower MRL for PYR described in NRCP 2013 (currently, for gamma and lambda-cyhalothrin).

Linearity, matrix effect, limit of quantification (LOQ), specificity, precision and trueness were carried out according to the European Commission guidelines [26]. Linearity was performed studying the regression significance and the linearity deviation of the matrix-matched calibration curves by analysis of variance, considering a *p* < 0.05 as significant. Additionally, the limit of detection was calculated by LOD ¼ 3.3 s/S, and the limit of quantification was calculated by LOQ ¼ 10 s/S, in which “s” was the standard deviation of the linear coefficient and “S” was the slope of the calibration curves. Analyte confirmation using GC-MS/MS was performed based on the ion ratio criteria in which the relative intensity of two transitions for each analyte shall correspond to those of the calibration standard, associated with analyte retention time and it was performed based on time-retention carried out in different columns 5% phenyl–methylpolysiloxane (OV-5, Ohio Valley, 15.0 m × 0.25 mm × 0.1 μm film thickness) and 5% phenyl-95% dimethyl polysiloxane (ZB5, Phenomenex, 30 m × 0.25 mm × 0.10 μm film thickness).

Selectivity and specificity were evaluated analyzing twenty different blank milk samples (fourteen raw milk samples, seven pasteurized milk) with and without addition of analytes, and analyzing samples spiked with the standard solutions at concentrations corresponding to VL. Method precision was calculated in terms of intra- and inter-day precision, expressed as coefficient of variation (CV%) and trueness in terms of average recovery for spike levels using three batches of eighteen blank samples spiked at 0.5, 1.0 and 1.5 × VL, prepared in three different days and analyzed independently.

## 3. Results and Discussion

### 3.1. Extraction Methodologies

The initial screening for extraction solvent optimization showed that ACN presents high efficiency for extracting PYR residues even in samples with high fat content as raw milk. This solvent possesses physicochemical properties that enable more selectivity to the extraction of moderately lipophilic compounds when LTP is used. Their melting point of approximately −45 °C allow the freezing of residual water content of the extract, reduce the solubility of polar to moderately polar interfering compounds (e.g., small proteins and lipoproteins) and promote the solidification of fat whilst the analytes remains in solution. The result is a smaller content of co-extractive material, as can be seen in the Table 3 and are in agreement with others authors [20,27]. Thus, ACN was chosen as extraction solvent. As well as low co-extractive material, a successful sample preparation procedure requires good recovery and adequate precision. So, modifications in LLE-LTP were tried (P05–P07) to improve recovery and the best results were observed in P07 whose presents satisfactory recovery (RE) and coefficient of variation (CV). 

**Table 3:** Recoveries obtained in screening evaluation of sample preparation method and in the optimized condition using Doehlert design study.

Sample Preparation Initial Screening				
Procedure ^a^	RE (%) ^b^	CV (%) ^c^	CoE (mg) ^d^	CE (abs) ^e^
P01 (MSPD)	50–60	35	8.0	0.455
P02 (HEX)	30–50	30	17.5	0.460
P03 (ACO)	50–60	25	15.4	0.508
P04 (EAC)	50–60	23	22.5	0.780
P05 (ACN)	50–60	22	5.1	0.320
P06 (ACN)	80–90	20	7.2	0.340
P07 (ACN)	80–90	20	7.4	0.335
**Doehlert Design**				
**Best Conditions ^f^**				
**Variable**	**(*t*/min)**	**(*V/*mL)**	**(*H/s)***	
**Value**	20.0	10.5	10.0	
**Equations ^g^**				
(l-CYH) *R%* = (1.55 *t)* + (6.07 *V)* − (1.20 *t*^2^) − (10.49 *V*^2^*)* − (0.58 *t V)*				
(PER) *R%* = (1.77 *t*) + (8.11 *V* ) + (−5.58 *t*^2^) − (14.97 *V* ^2^) + (4.12 *t V*)				
(g-CYH) *R%* = (1.40 *t)* + (6.07 *V)* − (1.2 *t*^2^) − (10.49 *V* ^2^*)* − (0.57 *t V)*				
(CYF) *R% = (0.82 t)* + (5.98 *V)* − (1.1 *t*^2^) − (9.49 *V* ^2^*)* − (0.5 *t V)*				
(CYP) *R%* = ( 0.88 *t*) + ( 6.38 *V*) − (−1.07 *t*^2^) − (9.33 *V* ^2^) − (−0.48 *t V*)				
(DEL) *R%* = (2.06 *t*) + (7.61 *V* ) + (0.87 *t*^2^) − (7.29 *V* ^2^) + (−0.09 *t V* ) + 2.00				
(FEV) *R%* = (1.09 *t*) + (−2.01 *t*^2^) + (−10.46 *V* ^2^) + (−0.23 *t V*)				

Notes: ^a^ Procedures coded in the screening, see Section 2.3 and Figure 2; ^b^ Range obtained for the seven PYR analyzed; ^c^ Coefficient of variation obtained for each procedure; ^d^ co-extractive material (CoE) evaluated by gravimetric measurement before reconstitution and injection; ^e^ *Trinder* method (505 nm, Analisa^®^ assay kit) used to co-extractive measurement (*n* = 6); ^f^ Final parameters optimized by *P07*; ^g^ Quadratic polynomials containing significant coefficients to recovery (*R%*) for each PYR.

Considering the properties of lipophilicity and adsorption in particulate phases founded at PYR, the inclusion of an additional operation (high-speed homogenizer) to disrupt protein-fatty globules was a right choice to overcome this interference [9,19]. Besides, high-speed homogenization associated with MgSO4 improved adsorption of water by salt because the comminution improved the contact surface between the particulates. This way, LTP step became more effective to trap co-extractives, dispensing a posterior SPE cleanup.

### 3.2. Doehlert Design

**Figure 3:**
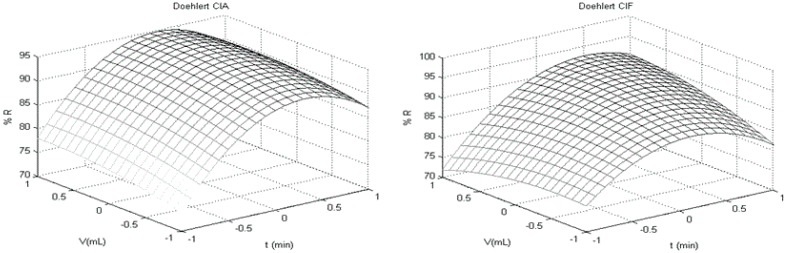

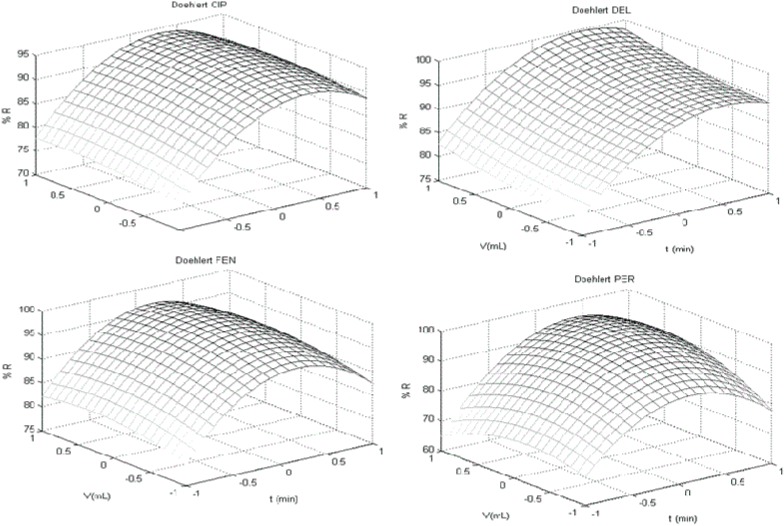
Response surfaces (RSMs) obtained using Doehlert design for recovery (%R) as response; volume of solvent (*V*) and time of agitation (*t*) were the significant factors obtained from modeling.

The first step of a multivariate optimization process consists in the choice of the most influent responses and factors. In this case, for procedure P07, the optimization was performed evaluating the volume of solvent (*V*), the contact time between sample and solvent during agitation on table (*t*) and the time of use of ultra-homogenizer (*H*). The response evaluated was analyte recovery (%R). The magnitude of influence these variables was evaluated considering the significance of polynomial degree regression (*p* > 0.95, Table 3) and inspection on response surfaces (Figure 3). 

The best adjustment for Doehlert design was found using quadratic equations with the homogenization time without significance over the modeling for all analytes. This way, the central point was considered as optimum conditions (better results for recovery) where the volume of ACN is 10.5 mL, ultra-homogenization time is 10 s and table agitation is 20 min. Moreover, when ACN volume for extraction exceeded 10.5 mL the recovery decrease probably because the co-extractive amount increase so much that analytes are trapped by protein-fatty globules during frozen (LTP step). The same occurs in table agitation when 20 min is exceeded leading analytes to be trapping by particulate matter.

### 3.3. Validation Procedure

The European Commission requires validation around the MRL values for authorized pesticides [26]. The validation levels used in this work were based on MRL applied in Brazilian National Residue Control Plan for CYH gamma e lambda isomers [28]. These pyrethroids have the lower MRL and consequently the method is adequate for the remaining PYR. The main challenge for a sample preparation procedure that will be performed in more than one detector systems (ECD and MS/MS) is the limited number of reagents compatible with ECD detector [21]. Despite the high sensibility and specificity of this detector, the presence of salts (such as NaCl), impurities or solvents that also contain halogens can produce significant instability in the chromatographic baseline and can, consequently, affect negatively both detection and quantification limits. Notwithstanding, the use of salts it is necessary in most of LLE-LTP procedures, for the more efficient cleanup because it is able to reduce the solubility of fat and protein in organic phase, besides to trap water. In this way, the use of MgSO_4_ allowed the validation for both detectors, due to the absence of halogens on their chemical composition, without significant difference (*t-*test performed for recovery, Table 4) and no need of an additional SPE step.

Satisfactory calibration curves (Table 5) with no significant deviation from linearity were obtained through all validation (*p* < 0.05, information no expressed). However, significant difference was found between solvent and matrix-matched curves (*p* < 0.05, information not expressed), for both detectors, with upper response for matrix-matched curves. This evidence corroborates other reports about matrix effects in GC analysis [21].

**Figure 4:**
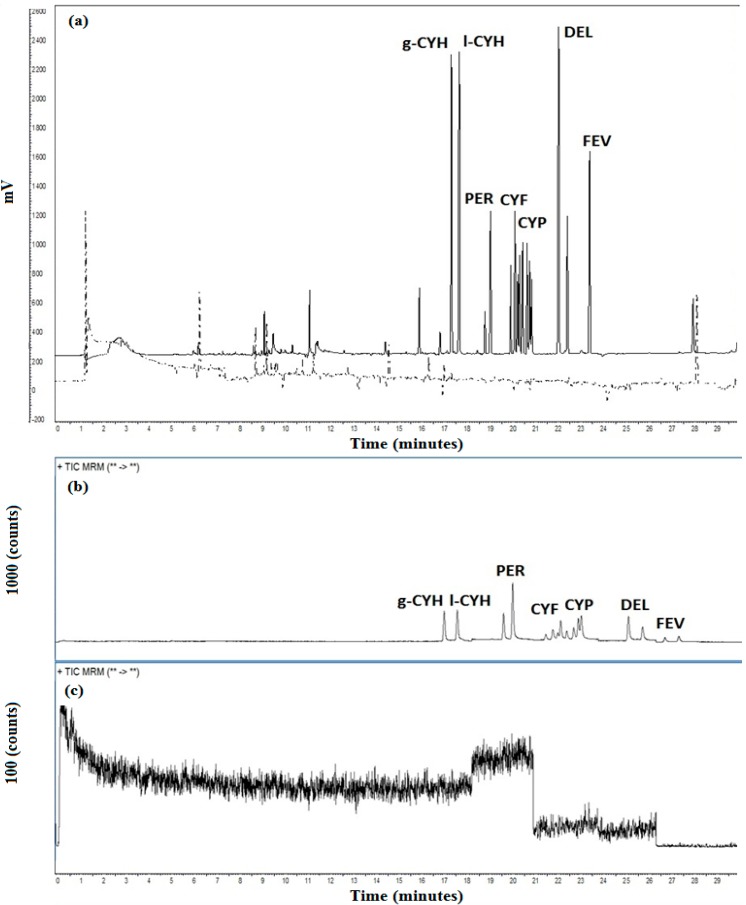
Comparison between chromatographic profiles for GC-ECD analyses (**a**) in spiked sample (solid line), blank milk (dotted line) and GC-MS/MS for spiked sample (**b**) and blank milk (**c**).

Despite of a positive matrix effect, the selectivity was evaluated analyzing an appropriate number of representative blank samples (*N* = 21) and checking for any interference *in* and *around* the retention time of the target analytes. As shown in Figure 4, no interfering compounds were observed in both GC-ECD and GC-MS/MS methods. The proposed sample preparation result in satisfactory analyte recovery for all analytes in the three different concentrations assessed. Method precision also met the validation criteria adopted (CV ≤ 20%) for intra-day, inter-day analyses and trueness (70%–120% to recovery range). These results are presented in the Table 5. 

**Table 4:** Comparison data between chromatographic systems.

Analyte	RT ^a^		RE (%) ^b^		
	MS/MS	ECD	MS/MS	ECD	*t*-value ^c^
CYH-g	20.74	17.43	97.5	86.8	−1.05
CYH-l	21.10	17.77	96.9	85.2	1.20
PER	22.63	18.91	97.7	88.9	0.89
CYF	23.96	20.22	93.28	91.49	0.56
CYP	24.53	20.56	96.67	85.15	1.60
DEL	25.83	22.14	99.95	92.85	0.80
FEV	27.23	23.14	95.50	88.50	0.76

Notes: ^a^; ^b^ Coefficient of regression obtained from matrix-matched calibration curves; ^c^ Limit of determination obtained from matrix-matched calibration curves; ^d^ Limit of quantification obtained from matrix-matched calibration curves; ^e^ Validation.

Electronic-impact ionization (EI) and subsequent product ion analysis (MRM mode) generally results in limits of quantification higher than those obtained with chemical ionization (CI) and, in some cases, ECD detector. This feature is related to peak separation (diastereoisomers) and low-mass ions (most of them with the same *m/z* ratios) [9]. Thus, it was necessary to improve the sensibility of the method based on GC-MS/MS by varying injection parameters. Cold splitless pulsed mode results in best performance for peak shape and signal/noise ratio. In this injection mode the injector is cooled to a temperature below the normal boiling point of the sample solvent (ACN). Theoretically, no vaporization occurs when the sample is injected using this injection mode. Once the syringe is removed from the inlet, the inlet is heated to vaporize the sample and transfer it to the column. The solvent vaporizes first and moves to column. Subsequently, analytes are vaporized and moves focused to the analytical column head. The main advantage is that analytes vaporize at lowest temperatures (near to their boiling point), rather than at a constant high temperature, minimizing thermal degradation while vapor pressure contributes to improve peak shape and allow a wide range of analytes to vaporize. After product ion analysis using different parent ions and collision energy optimization (CE) for final transitions, optimized values can be seen at Table 6 (all transitions showed ion ratio variation lower than 20%).

**Table 5:** Summary of validation parameters.

*GC-ECD*												
				0.5 VL ^e^			1.0 VL			1.5 VL		
Analyte ^a^	R^2^^b^	LD ^c^	LQ ^d^	RE (%) ^f^	CV (%) ^g^	CV (%) ^h^	RE (%)	CV (%)	CV (%)	RE (%)	CV (%)	CV (%)
CYH-g(25)(2)	0.99	0.3	0.9	95.4	12.3	10.4	92.1	8.5	13.7	96.5	8.3	12.5
CYH-l (25)	0.99	0.3	0.9	93.1	10.6	11.6	96.5	9.6	12.6	94.3	9.6	13.6
PER (50)	0.99	0.4	1.1	95.7	14.5	15.1	93.4	16.5	17.8	90.9	10.8	15.2
CYF (40)	0.99	0.7	2.1	90.8	8.6	14.9	87.6	9.6	12.3	92.0	9.2	9.4
CYP (100)	0.99	0.6	1.8	88.7	10.8	13.7	90.6	8.1	10.3	95.3	12.1	14.5
DEL (30)	0.99	0.7	2.1	95.5	8.9	15.2	92.2	9.5	12.7	90.4	8.1	11.7
FEV (40)	0.99	0.7	2.1	99.6	12.1	10.5	101.4	11.9	14.7	97.4	12.5	14.2
***GC-MS/MS***												
				**0.5 VL**			**1.0 VL**			**1.5 VL**		
**Analyte**	**R^2^**	**LD**	**LQ**	**RE(%)**	**CV (%)**	**CV (%)**	**RE (%)**	**CV(%)**	**CV (%)**	**RE (%)**	**CV (%)**	**CV (%)**
CYH-g	0.99	0.3	0.9	86.5	8.5	9.6	88.0	14.5	15.1	90.2	9.0	13.7
CYH-l	0.99	0.3	0.9	84.2	9.0	10.2	90.2	11.3	12.0	85.6	10.6	14.6
PER	0.99	0.4	1.2	88.9	9.7	12.4	93.6	12.8	12.7	92.3	11.2	12.5
CYF	0.99	0.7	2.2	90.3	13.5	13.2	90.9	9.6	11.8	94.5	8.1	9.0
CYP	0.99	0.7	2.2	88.4	12.9	14.0	87.3	8.2	10.9	89.1	10.5	11.0
DEL	0.99	0.9	2.7	85.4	11.5	11.9	93.7	13.5	15.4	90.2	15.5	15.6
FEV	0.99	1.0	3.0	88.9	13.6	15.8	91.6	14.7	16.6	92.0	12.0	13.9

Notes: ^a^ Values between brackets are the Maximum Limit Residue (μg·L^−1^) established at NRCP 2013; ^b^ Coefficient of regression obtained from matrix-matched calibration curves; ^c^ Limit of determination obtained from matrix-matched calibration curves; ^d^ Limit of quantification obtained from matrix-matched calibration curves; ^e^ Validation level (VL) adopted at 25 μg·L^−1^; ^f^ Recovery expressed as the average of all samples from intra and inter-day experiments; ^g^ Intra-day precision expressed as coef-ficient of variation; ^h^ Inter-day precision expressed as coefficient of variation.

**Table 6:** Mass spectrometry settings for GC-MS/MS analysis of pyrethroids in MRM mode.

Analytes	Quantifier ^a^			Qualifier ^a^		
	Precursorion (m/z)	Production (m/z)	CE (eV) ^b^	Precursorion (m/z)	Production (m/z)	CE (eV)
CYH-g	181.1	127.1	30	181.1	152.1	35
CYH-l	180.0	127.1	30	181.1	152.1	35
PER	183.3	165.1	10	183.3	168.1	15
CYF	181.1	152.1	5	181.1	127.1	5
CYP	163.0	127.1	5	163.0	91.1	5
DEL	166.9	125.0	5	124.9	89.0	5
FEV	252.8	173.9	5	252.8	172.0	5

Note: ^a^ Transitions used for quantification and complementar (*qualifier*) identification; ^b^Collision energy.

### 3.4. Method Applicability

After validation, the proposed method was applied to the simultaneous analysis of PYR residues in 50 real samples of bovine raw milk obtained from investigations realized during 2013. All samples showed compliant results, *i.e.*, PYR levels lower than MRL. Notwithstanding, several samples presented levels of PYR: 49 samples presented levels of deltamethrin at concentrations between LD and LQ; in five samples, deltamethrin was detected at concentrations above LQ but below MRL. CYP was determined in just one sample also at a concentration between LD and LQ. Results show that the method can be easily performed to PYR routine analysis with a high degree of confidence.

## 4. Conclusions

Despite the fact that several chemometric tools are currently available to use in analytical chemistry, their use is less frequent than expected. Herein, we describe the development, optimization and validation of two methods for determination of PYR residues in milk samples. Sample preparation technique was deeply optimized, firstly by an experiment to solvent selection, followed by a statistical optimization procedure using a Doehlert experimental design. Albeit LLE-LTP have been previously applied by our own research group for veterinary drugs residues analysis and by other authros to PYR analysis, the modifications introduced in LLE-LTP procedure showed to be more efficient and less expensive than the other procedures by LLE with range of different sorbents that can increase the variability and the cost for analysis. Moreover, to the best of our knowledge, a sample preparation method for PYR determination in milk samples extensively optimized has never been reported to date. The use of magnesium salt in cleanup procedure turn the method into an appropriate approach for both GC techniques, permitting the parallel analysis of the same sample in more than one analytical system simultaneously. Methods were fully validated according the SANCO guidelines and showed satisfactory responses for all evaluated parameters. The method was also applied to real samples in routine analysis. Ergo, the present method is able to be applied as screening, quantitative and confirmatory method for determination of PYR residues in milk in the context of the National Residues Control Plan.
